# Supplementary material for: Connectivity of Fennoscandian Shield terrestrial deep biosphere microbiomes with surface communities
Source: Commun Biol. 2022 Jan 11;5:37. doi: 10.1038/s42003-021-02980-8 (PMC8752596; doi:10.1038/s42003-021-02980-8)
Supplement: Supplementary file 2 — Supplementary Materials [file 42003_2021_2980_MOESM2_ESM.pdf]

Connectivity of Fennoscandian Shield terrestrial deep biosphere microbiomes with surface communities

George Westmeijer, Maliheh Mehrshad, Stephanie Turner, Linda Alakangas, Varvara Sachpazidou, Carina Bunse, Jarone Pinhassi, Marcelo Ketzer, Mats Åström, Stefan Bertilsson, & Mark Dopson

Supplementary Materials

Table S1. Data sources of the 16S rRNA gene amplicon sequences. Either a NCBI project reference or a European Nucleotide Archive (ENA) reference is provided.

Environment	WGS coordinates (lat, lon)	# of samples	Total # replicates	ENA[a]/NCBI[b] project number	DOI
Baltic surface seawater	56°55.854', 17°3.642'	93	93	PRJEB40890[a]	This study
Baltic benthic seawater	57°53.214', 16°35.934' 57°53.531', 16°35.165'	3	10	PRJNA323408[b] PRJNA347538[b]	10.1007/s00248-018-1229-6 10.3389/fmicb.2017.02453
Upper sediment	57°53.531', 16°35.165' 57°53.214', 16°35.934' 57°53.214', 16°35.934'	6	19	PRJNA347538[b] PRJNA323408[b] PRJNA322450[b]	10.3389/fmicb.2017.02453 10.1007/s00248-018-1229-6 10.1186/s40168-017-0311-5
Lower sediment	57°53.214', 16°35.934'	7	14	PRJEB41312[a]	This study
Upper soil groundwater	57°24.7622', 16°37.7207'	3	9	PRJEB41312[a]	This study
Lower soil groundwater	57°25.7702', 16°41.3716'	1	3	PRJEB41312[a]	This study
Meteoric groundwater	57°26.0666', 16°39.6'	1	3	PRJEB41312[a]	This study
Modern marine groundwater	57°26.0666', 16°39.6'	16	48	PRJNA434543[b]	10.3389/fmicb.2018.02880
Old saline groundwater	57°26.0666', 16°39.6'	5	15	PRJNA434543[b]	10.3389/fmicb.2018.02880

Table S2. Sampling details. Dates and DNA extraction details of sampling efforts carried out for this study.

SKB ID	Sample ID	Sampling date (dd/mm/yyyy)	Total water / sediment volume (L)	DNA extract concentration (ng/μL)
**Baltic surface seawater**				
---	P1313_2001 to P1313_2096	20/04/2011 - 11/12/2013	3.0 (per sample)	0.38 - 15.9
**Baltic lower sediment**				
---	P11852_1047	18/06/2018	10 mL	28.6
---	P11852_1048	18/06/2018	10 mL	13.5
---	P11852_1049	18/06/2018	10 mL	13.5
---	P11852_1050	18/06/2018	10 mL	19.8
---	P11852_1051	18/06/2018	10 mL	17.9
---	P11852_1052	18/06/2018	10 mL	6.28
---	P11852_1053	18/06/2018	10 mL	19.0
---	P11852_1054	18/06/2018	10 mL	26.0
---	P11852_1056	18/06/2018	10 mL	30.3
---	P11852_1057	18/06/2018	10 mL	9.08
---	P11852_1058	18/06/2018	10 mL	11.2
---	P11852_1059	18/06/2018	10 mL	7.61
---	P11852_1060	18/06/2018	10 mL	9.30
---	P11852_1061	18/06/2018	10 mL	4.95
**Äspö Island upper soil groundwater**				
**SSM215**	P15156_1054	20/11/2019	0.45	10.3
**SSM215**	P15156_1055	20/11/2019	0.48	11.4
**SSM215**	P15156_1056	20/11/2019	0.46	11.5
**SSM42**	P15156_1064	27/11/2019	1.0	14.9
**SSM42**	P15156_1065	27/11/2019	1.0	41.5
**SSM42**	P15156_1066	27/11/2019	1.0	46.5
**SSM268**	P15156_1067	27/11/2019	1.0	16.6
**SSM268**	P15156_1068	27/11/2019	1.0	10.7
**SSM268**	P15156_1069	27/11/2019	1.0	9.22
**Äspö Island lower soil groundwater**				
**SSM22**	P15156_1057	20/11/2019	0.54	3.82
**SSM22**	P15156_1058	20/11/2019	0.58	3.51
**SSM22**	P15156_1059	20/11/2019	0.60	1.35
**Meteoric groundwater**				
**KR0015B**	P15009_1057	21/10/2019	48.1	56.0
**KR0015B**	P15009_1059	25/10/2019	60.8	> 60
**KR0015B**	P15009_1060	28/10/2019	57.5	> 60

Table S3. 16S rRNA gene amplicon sequencing details. Number of reads per sample including the total number of sequences, quality control, and number of ASVs per sample throughout the DADA2 pipeline.

Sample	Environment	Number of raw reads (x 1000)	Retained quality filter (%)	Number of ASVs
P1313_2001	Surface seawater	183.1	95.5	519
P1313_2002	Surface seawater	153.5	87.8	459
P1313_2003	Surface seawater	28.2	100.0	203
P1313_2004	Surface seawater	58.9	99.6	368
P1313_2005	Surface seawater	55.7	99.3	293
P1313_2006	Surface seawater	137.0	94.0	506
P1313_2007	Surface seawater	87.2	99.4	377
P1313_2008	Surface seawater	4.0	99.8	87
P1313_2009	Surface seawater	125.4	94.6	475
P1313_2010	Surface seawater	91.7	99.7	363
P1313_2011	Surface seawater	206.8	98.3	667
P1313_2012	Surface seawater	109.1	97.9	344
P1313_2013	Surface seawater	259.9	98.0	659
P1313_2014	Surface seawater	125.5	97.8	545
P1313_2015	Surface seawater	101.9	99.0	505
P1313_2016	Surface seawater	66.3	98.3	387
P1313_2017	Surface seawater	116.9	99.9	373
P1313_2018	Surface seawater	267.1	100.0	453
P1313_2019	Surface seawater	144.9	99.9	395
P1313_2020	Surface seawater	26.9	99.9	248
P1313_2021	Surface seawater	19.4	99.7	220
P1313_2022	Surface seawater	54.6	99.7	316
P1313_2023	Surface seawater	36.9	99.6	299
P1313_2024	Surface seawater	165.3	99.8	406
P1313_2025	Surface seawater	75.8	99.7	431
P1313_2026	Surface seawater	10.8	99.0	208
P1313_2027	Surface seawater	50.3	97.0	427
P1313_2028	Surface seawater	15.0	98.3	220
P1313_2029	Surface seawater	139.6	98.0	556
P1313_2030	Surface seawater	56.5	98.1	378
P1313_2031	Surface seawater	39.5	95.9	367
P1313_2032	Surface seawater	50.0	98.6	381
P1313_2033	Surface seawater	191.8	89.7	764
P1313_2034	Surface seawater	122.1	89.0	705
P1313_2035	Surface seawater	117.8	95.1	824
P1313_2036	Surface seawater	5.0	98.9	71
P1313_2037	Surface seawater	181.5	97.6	532
P1313_2038	Surface seawater	24.8	94.4	78
P1313_2039	Surface seawater	68.1	97.9	300
P1313_2040	Surface seawater	152.0	95.8	443
P1313_2041	Surface seawater	1.7	98.3	93
P1313_2042	Surface seawater	8.0	100.0	70
P1313_2043	Surface seawater	52.7	91.8	280
P1313_2044	Surface seawater	124.9	91.8	316
P1313_2045	Surface seawater	87.3	97.4	424
P1313_2046	Surface seawater	11.2	96.7	252
P1313_2047	Surface seawater	196.8	92.5	498
P1313_2048	Surface seawater	203.4	93.1	663
P1313_2049	Surface seawater	84.3	97.9	564
P1313_2050	Surface seawater	9.8	96.9	178
P1313_2051	Surface seawater	106.8	98.0	375
P1313_2052	Surface seawater	115.8	97.6	389
P1313_2053	Surface seawater	72.9	99.1	450
P1313_2055	Surface seawater	91.5	97.6	431
P1313_2056	Surface seawater	139.6	97.9	425
P1313_2057	Surface seawater	24.9	98.5	331
P1313_2058	Surface seawater	9.6	98.3	216
P1313_2059	Surface seawater	31.7	98.9	317
P1313_2060	Surface seawater	90.6	99.3	456
P1313_2061	Surface seawater	22.3	98.5	222
P1313_2062	Surface seawater	115.2	91.3	649
P1313_2063	Surface seawater	21.5	93.2	292
P1313_2064	Surface seawater	130.1	97.8	597
P1313_2065	Surface seawater	80.5	94.4	184
P1313_2066	Surface seawater	90.3	94.1	342
P1313_2067	Surface seawater	263.8	99.7	297
P1313_2068	Surface seawater	129.5	96.6	399
P1313_2069	Surface seawater	53.2	95.2	309

P1313_2070	Surface seawater	102.8	97.0	424
P1313_2071	Surface seawater	101.4	96.4	446
P1313_2072	Surface seawater	103.3	99.7	490
P1313_2074	Surface seawater	283.2	99.8	559
P1313_2075	Surface seawater	118.7	99.6	594
P1313_2076	Surface seawater	43.1	99.9	237
P1313_2077	Surface seawater	48.3	98.1	464
P1313_2078	Surface seawater	91.9	97.2	411
P1313_2079	Surface seawater	123.3	80.6	631
P1313_2080	Surface seawater	142.8	97.0	449
P1313_2081	Surface seawater	7.0	95.8	198
P1313_2082	Surface seawater	303.7	81.9	757
P1313_2083	Surface seawater	36.4	97.3	335
P1313_2084	Surface seawater	149.6	95.5	547
P1313_2085	Surface seawater	275.6	96.3	820
P1313_2086	Surface seawater	39.7	94.8	462
P1313_2087	Surface seawater	20.5	96.3	333
P1313_2088	Surface seawater	225.6	97.9	720
P1313_2089	Surface seawater	125.8	97.4	587
P1313_2090	Surface seawater	1.3	98.4	46
P1313_2091	Surface seawater	8.9	94.7	133
P1313_2092	Surface seawater	257.2	98.3	395
P1313_2094	Surface seawater	547.1	99.5	566
P1313_2095	Surface seawater	101.0	97.3	526
P1313_2096	Surface seawater	316.8	88.0	904
E10	Benthic seawater	179.5	94.2	1877
E11	Benthic seawater	208.0	74.6	1583
E12	Benthic seawater	200.0	93.9	1854
E19	Benthic seawater	102.6	93.9	511
E20	Benthic seawater	74.7	91.0	872
E21	Benthic seawater	69.9	93.7	588
E26	Benthic seawater	52.5	94.6	402
E27	Benthic seawater	53.7	96.1	363
E28	Benthic seawater	35.5	96.4	296
E29	Benthic seawater	43.9	95.5	333
E01	Upper sediment	23.8	96.4	1374
E02	Upper sediment	7.0	98.6	465
E03	Upper sediment	27.0	97.7	1560
E04	Upper sediment	13.7	86.8	694
E05	Upper sediment	3.4	86.1	240
E06	Upper sediment	4.0	98.6	286
E07	Upper sediment	1.7	93.2	133
E08	Upper sediment	3.9	97.9	284
E09	Upper sediment	14.3	99.9	855
E13	Upper sediment	66.0	82.2	1052
E14	Upper sediment	13.3	98.2	291
E15	Upper sediment	128.0	96.5	899
E16	Upper sediment	71.2	87.7	2176
E17	Upper sediment	32.2	91.5	1320
E18	Upper sediment	80.0	89.0	2396
E22	Upper sediment	63.2	99.0	1874
E23	Upper sediment	33.5	97.1	1071
E24	Upper sediment	61.9	98.6	1875
E25	Upper sediment	41.2	99.3	1195
P11852_1047	Lower sediment	17.2	90.7	1019
P11852_1048	Lower sediment	77.8	99.1	2654
P11852_1049	Lower sediment	29.3	59.5	983
P11852_1050	Lower sediment	31.5	62.8	1078
P11852_1051	Lower sediment	10.0	68.1	428
P11852_1052	Lower sediment	40.4	91.9	1891
P11852_1053	Lower sediment	39.2	91.0	2022
P11852_1054	Lower sediment	17.6	93.4	1006
P11852_1056	Lower sediment	86.2	94.4	3093
P11852_1057	Lower sediment	46.4	98.2	2205
P11852_1058	Lower sediment	122.3	89.6	4258
P11852_1059	Lower sediment	51.5	96.1	2177
P11852_1060	Lower sediment	64.3	95.6	2693
P11852_1061	Lower sediment	83.4	99.4	2687
P15156_1054	Upper soil	18.7	99.8	1550
P15156_1055	Upper soil	12.7	99.6	1062
P15156_1056	Upper soil	78.3	99.8	5119
P15156_1064	Upper soil	10.9	100.0	703
P15156_1065	Upper soil	12.3	99.8	737
P15156_1066	Upper soil	17.8	100.0	999
P15156_1067	Upper soil	25.0	100.0	1851
P15156_1068	Upper soil	80.4	100.0	4392
P15156_1069	Upper soil	71.0	99.9	4003

P15156_1057	Lower soil	114.0	100.0	3346
P15156_1058	Lower soil	20.1	100.0	1038
P15156_1059	Lower soil	89.1	100.0	2675
P15009_1057	Meteoric	75.6	100.0	1260
P15009_1059	Meteoric	12.1	99.8	390
P15009_1060	Meteoric	54.6	100.0	893
QK1636-S08	Modern marine	112.2	100.0	1227
QK1636-S12	Modern marine	60.7	99.9	741
QK1636-S20	Modern marine	91.2	100.0	1275
QK1636-S24	Modern marine	150.5	99.8	1582
QK1636-S32	Modern marine	159.2	100.0	1084
QK1636-S36	Modern marine	139.8	99.9	1305
QK1636-S37	Modern marine	24.5	99.9	594
QK1636-S38	Modern marine	61.0	100.0	765
QK1636-S39	Modern marine	46.6	99.9	867
QK1636-S43	Modern marine	33.3	100.0	503
QK1636-S44	Modern marine	46.7	100.0	534
QK1636-S45	Modern marine	57.4	100.0	457
QK1636-S46	Modern marine	30.7	100.0	667
QK1636-S47	Modern marine	47.1	100.0	910
QK1636-S48	Modern marine	58.5	100.0	933
QK1636-S49	Modern marine	170.2	100.0	931
QK1636-S50	Modern marine	222.9	100.0	1217
QK1636-S51	Modern marine	56.4	100.0	603
QK1636-S55	Modern marine	45.5	100.0	845
QK1636-S56	Modern marine	40.1	100.0	779
QK1636-S57	Modern marine	52.7	100.0	781
QK1636-S58	Modern marine	93.5	100.0	835
QK1636-S59	Modern marine	39.8	100.0	521
QK1636-S60	Modern marine	88.8	100.0	686
QK1636-S61	Modern marine	96.4	100.0	545
QK1636-S62	Modern marine	138.9	100.0	655
QK1636-S63	Modern marine	227.1	100.0	754
QK1636-S64	Modern marine	70.3	100.0	686
QK1636-S65	Modern marine	101.6	100.0	839
QK1636-S66	Modern marine	115.1	100.0	861
QK1636-S67	Modern marine	28.3	100.0	495
QK1636-S68	Modern marine	63.8	100.0	621
QK1636-S69	Modern marine	44.7	100.0	684
QK1636-S70	Modern marine	112.5	100.0	772
QK1636-S71	Modern marine	182.2	100.0	808
QK1636-S72	Modern marine	121.5	100.0	716
QK1636-S73	Modern marine	164.2	100.0	1201
QK1636-S74	Modern marine	197.1	100.0	1222
QK1636-S75	Modern marine	41.8	100.0	667
QK1636-S76	Modern marine	24.4	100.0	524
QK1636-S77	Modern marine	24.4	100.0	515
QK1636-S78	Modern marine	42.2	100.0	764
QK1636-S79	Modern marine	65.7	100.0	925
QK1636-S80	Modern marine	58.4	100.0	860
QK1636-S81	Modern marine	59.8	100.0	763
QK1636-S88	Modern marine	59.2	100.0	1147
QK1636-S89	Modern marine	24.1	100.0	656
QK1636-S90	Modern marine	28.0	100.0	752
QK1636-S04	Old saline	121.4	100.0	516
QK1636-S16	Old saline	95.6	100.0	561
QK1636-S28	Old saline	196.0	100.0	506
QK1636-S40	Old saline	97.6	100.0	920
QK1636-S41	Old saline	125.2	100.0	960
QK1636-S42	Old saline	152.3	100.0	1013
QK1636-S52	Old saline	106.6	100.0	746
QK1636-S53	Old saline	91.7	100.0	688
QK1636-S54	Old saline	135.0	100.0	986
QK1636-S82	Old saline	298.9	100.0	1113
QK1636-S83	Old saline	53.3	100.0	511
QK1636-S84	Old saline	20.0	100.0	354
QK1636-S85	Old saline	23.1	100.0	397
QK1636-S86	Old saline	45.1	100.0	704
QK1636-S87	Old saline	54.1	100.0	680

Table S4. Output of alpha diversity statistical testing. Values on the main diagonal are the mean Shannon index within each environment ± the standard deviation among the samples. A one-way ANOVA revealed a difference of diversity among the environments ($p = 1.76e^{-10}$). Post-hoc testing was performed using Tukey's HSD test. Significance codes: *** 0.001 ** 0.01 * 0.05 refer to Tukey's adjusted p-values, correcting for multiple comparisons.

	**Baltic surface seawater**	**Baltic benthic seawater**	**Upper sediment**	**Lower sediment**	**Upper soil groundwater**	**Lower soil groundwater**	**Meteoric groundwater**	**Modern marine groundwater**	**Old saline groundwater**
**Baltic surface seawater**	4.1 ± 0.12	***	***	***	***	***	*	**	
**Baltic surface seawater**		4.9 ± 0.28	***	***	***	**		***	*
**Upper sediment**			5.9 ± 0.84		*			***	***
**Lower sediment**				6.7 ± 0.38			**	***	***
**Upper soil groundwater**					6.6 ± 1.0		*	***	***
**Lower soil groundwater**						6.3 ± 0.0		***	***
**Meteoric groundwater**							5.2 ± 0.0	***	**
**Modern marine groundwater**								3.7 ± 0.97	
**Old saline groundwater**									3.9 ± 0.50

Table S5. Output of beta diversity statistical testing. PERMANOVA R^2 values on a Bray-Curtis dissimilarity matrix (top diagonal) estimating the environments' effect on the samples with a value close to 1 indicative of a high degree of clustering based on the environment (significance codes: *** 0.001 ** 0.01 * 0.05).

	Baltic surface seawater	Baltic benthic seawater	Upper sediment	Lower sediment	Upper soil groundwater	Lower soil groundwater	Meteoric groundwater	Modern marine groundwater	Old saline groundwater
**Baltic surface seawater**		0.04 *	0.12 *	0.15 *	0.09 *	0.05 *	0.05 *	0.21 *	0.14 *
**Baltic surface seawater**			0.20 *	0.37 *	0.29 *	0.37	0.35	0.17 *	0.31 *
**Upper sediment**				0.20 *	0.14 *	0.14	0.13	0.14 *	0.19 *
**Lower sediment**					0.26 *	0.28 *	0.27	0.19 *	0.31 *
**Upper soil groundwater**						0.24	0.23	0.11 *	0.21 *
**Lower soil groundwater**							0.48	0.08 *	0.22
**Meteoric groundwater**								0.07 *	0.19
**Modern marine groundwater**									0.07 *
**Old saline groundwater**									

Table S6a. Number of ASVs shared between the various environments. The number of ASVs within each environment are displayed along the center diagonal (in bold).

	Baltic surface seawater	Baltic benthic seawater	Upper sediment	Lower sediment	Upper soil groundwater	Lower soil groundwater	Meteoric groundwater	Modern marine groundwater	Old saline groundwater
**Baltic surface seawater**	**5452**	909	664	198	102	42	6	86	79
**Baltic benthic seawater**		**4340**	2093	882	67	41	12	125	91
**Upper sediment**			**6899**	1472	62	52	15	141	109
**Lower sediment**				**10349**	27	57	20	218	98
**Upper soil groundwater**					**11882**	142	47	50	52
**Lower soil groundwater**						**4154**	227	378	334
**Meteoric groundwater**							**1660**	264	209
**Modern marine groundwater**								**9315**	2186
**Old saline groundwater**									**3408**

Table S6b. Abundance of the shared ASVs among the various environments. The abundance is displayed as relative abundance that sums up to 1 within each environment.

	Baltic surface seawater	Baltic benthic seawater	Upper sediment	Lower sediment	Upper soil groundwater	Lower soil groundwater	Meteoric groundwater	Modern marine groundwater	Old saline groundwater
**Baltic surface seawater**		0.89	0.81	0.30	0.39	0.23	0.00	0.02	0.02
**Baltic benthic seawater**	0.85		0.88	0.19	0.32	0.14	0.00	0.03	0.03
**Upper sediment**	0.21	0.73		0.39	0.03	0.02	0.00	0.07	0.05
**Lower sediment**	0.08	0.37	0.43		0.01	0.01	0.00	0.04	0.02
**Upper soil groundwater**	0.01	0.01	0.01	0.00		0.09	0.01	0.01	0.01
**Lower soil groundwater**	0.02	0.02	0.03	0.02	0.21		0.10	0.18	0.17
**Meteoric groundwater**	0.00	0.05	0.10	0.01	0.19	0.22		0.36	0.35
**Modern marine groundwater**	0.02	0.34	0.33	0.18	0.03	0.13	0.22		0.92
**Old saline groundwater**	0.06	0.22	0.23	0.19	0.06	0.11	0.29	0.98	

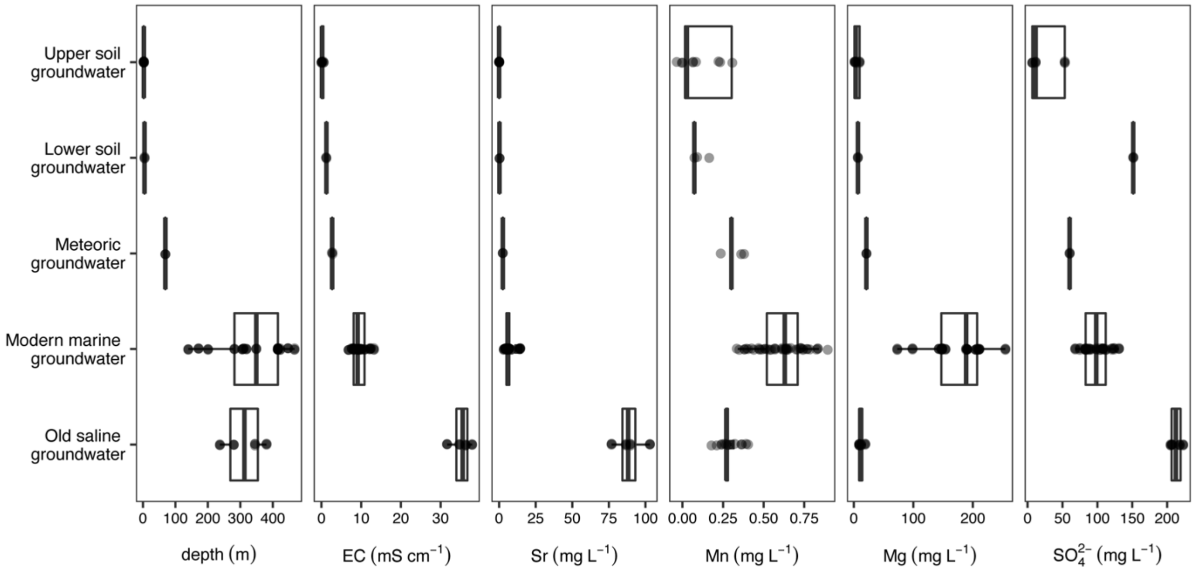

Fig. S1. Boxplot depicting depth and chemistry of the soil plus deep biosphere groundwaters.

EC; electrical conductivity.

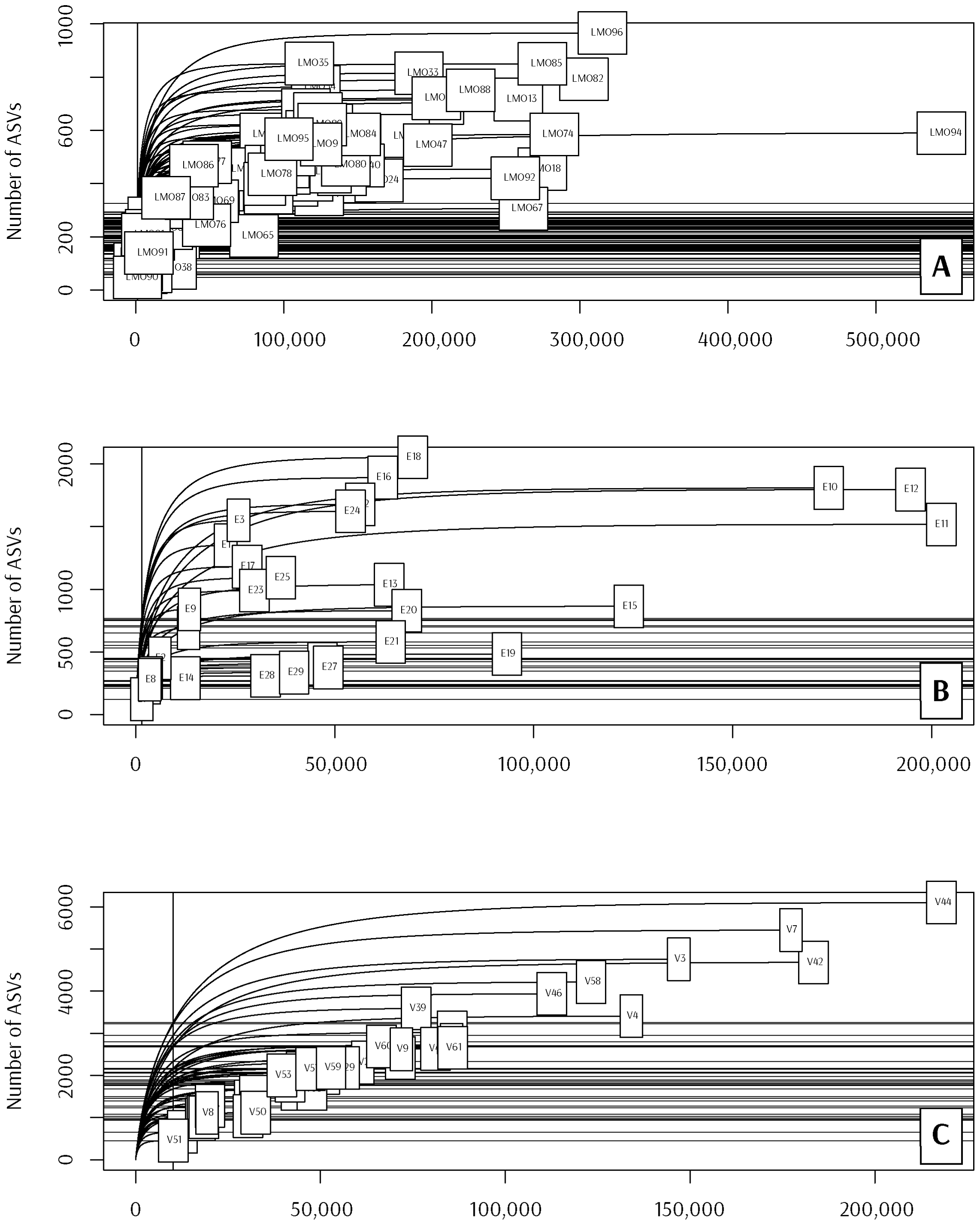

A
Number of ASVs
0
200
600
1000
0
100,000
200,000
300,000
400,000
500,000
LMO96
LMO35
LMO33
LMO85
LMO82
LMO88
LMO13
LMO94
LMO74
LMO84
LMO95
LMO47
LMO86
LMO78
LMO80
LMO92
LMO18
LMO87
LMO83
LMO69
LMO67
LMO76
LMO65
LMO91
B
Number of ASVs
0
500
1000
2000
0
50,000
100,000
150,000
200,000
E18
E16
E10
E12
E24
E3
E11
E17
E25
E13
E23
E20
E15
E9
E21
E19
E8
E14
E28
E29
E27
C
Number of ASVs
0
2000
4000
6000
0
50,000
100,000
150,000
200,000
V44
V7
V3
V42
V58
V46
V39
V4
V60
V9
V61
V53
V59
V8
V50
V51

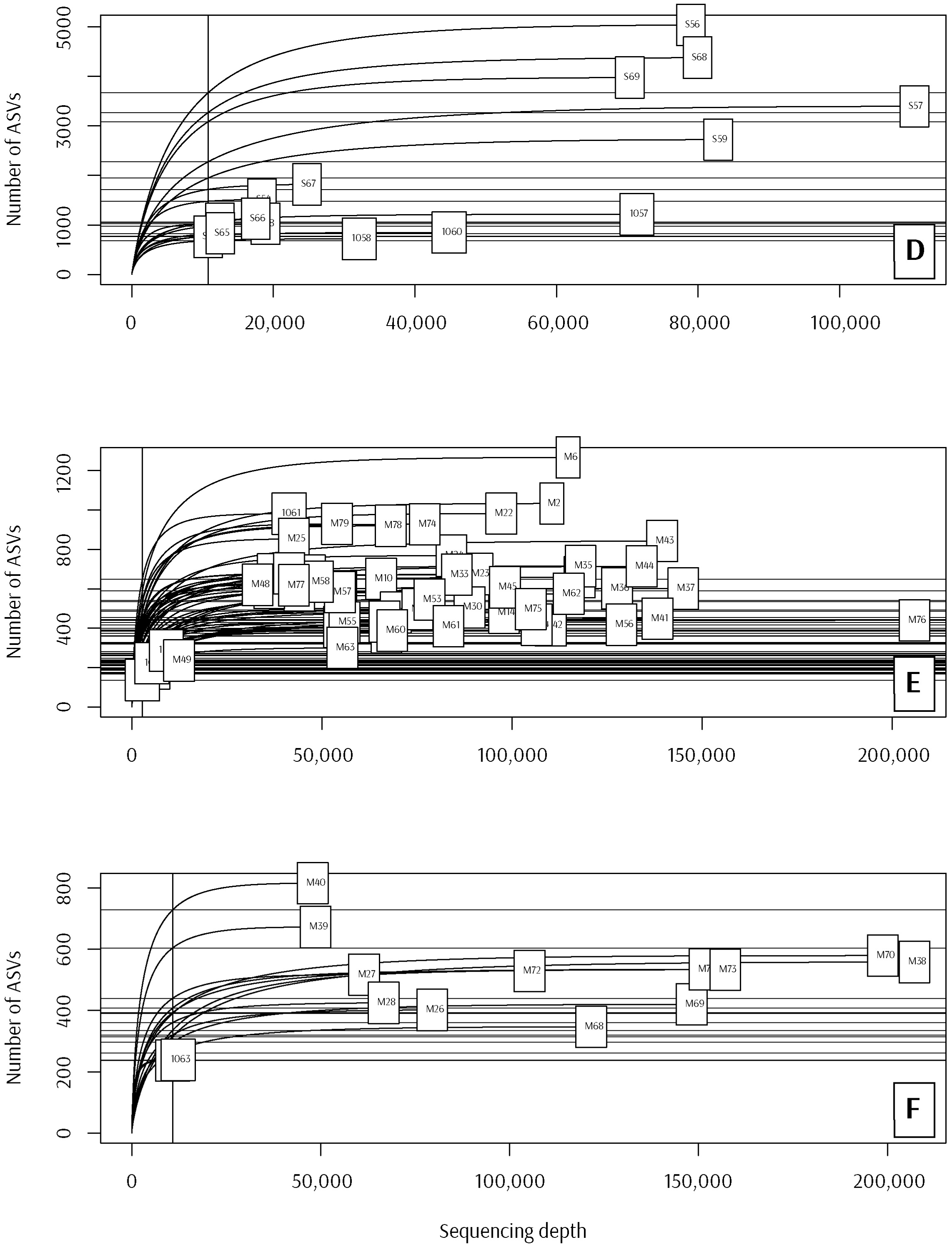

Fig. S2. Rarefaction curves depicting the relation between sequencing depth and ASV count. The sequencing depth (X-axis) is expressed as the number of reads. (**A**) Baltic surface seawater; (**B**) Baltic benthic seawater and upper sediment; (**C**) lower sediment; (**D**) upper and lower soil groundwater plus meteoric groundwater; (**E**) modern marine groundwater; and (**F**) old saline groundwater.

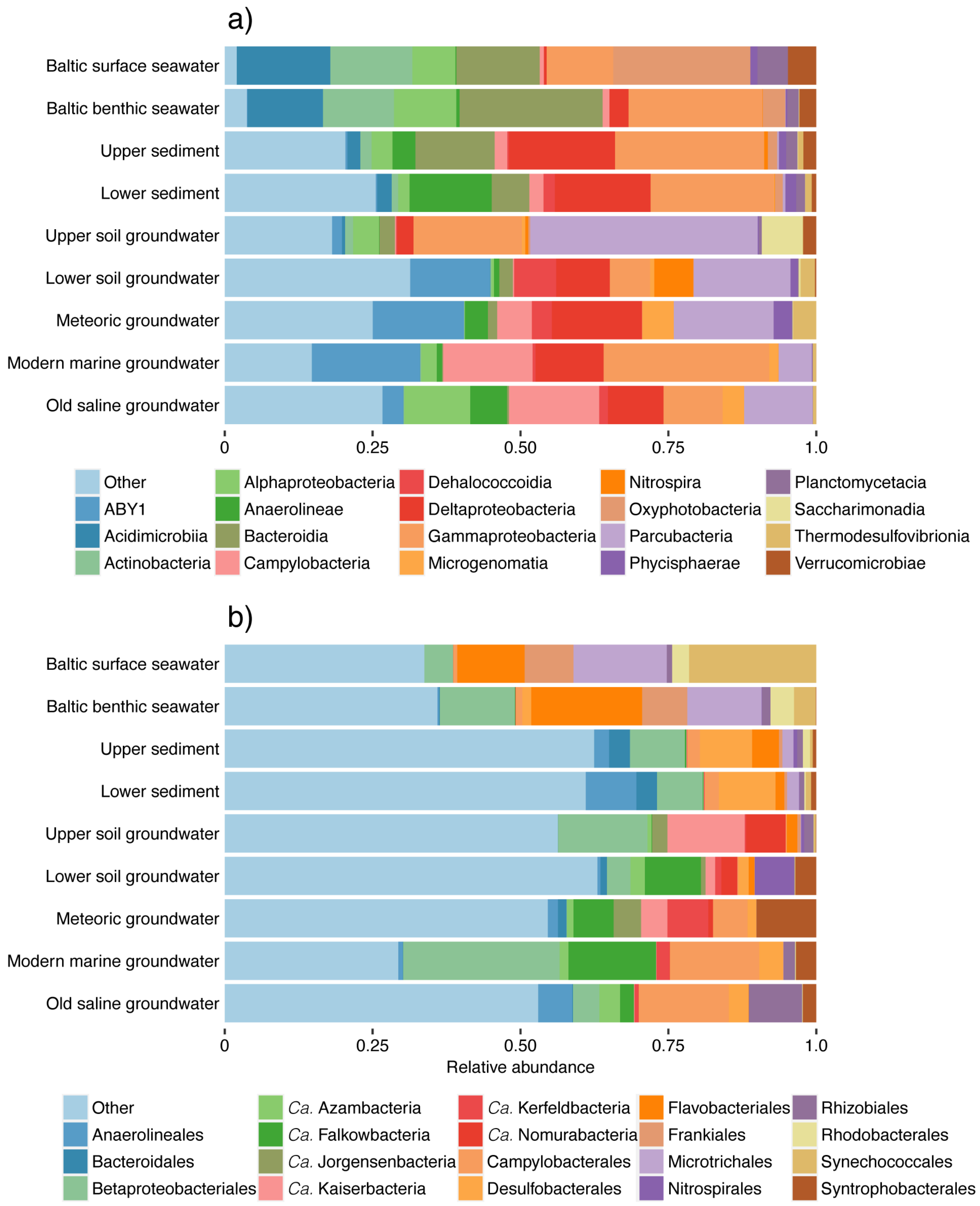

Fig. S3. Bar plot of the microbial communities sorted at the taxonomic level of class (a) and order (b). The figures depict the 19 most abundant groups over all the environments with the remaining taxa grouped as "Other".

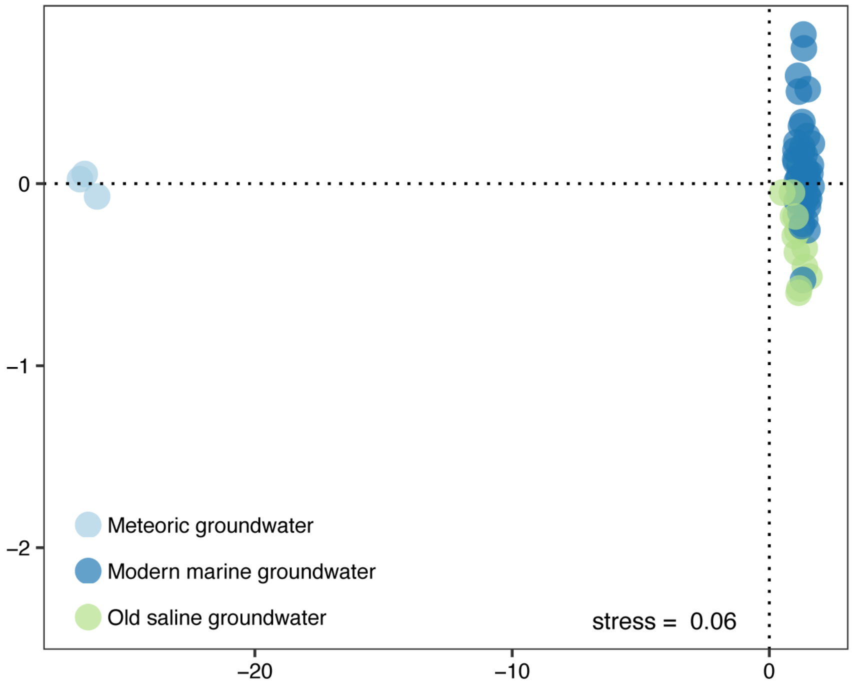

Fig. S4. Ordination plot of microbial communities from deep biosphere groundwaters. A Bray-Curtis dissimilarity matrix on the ASVs from the various environments was used as input for nonmetric multi-dimensional scaling (NMDS) as ordination method.

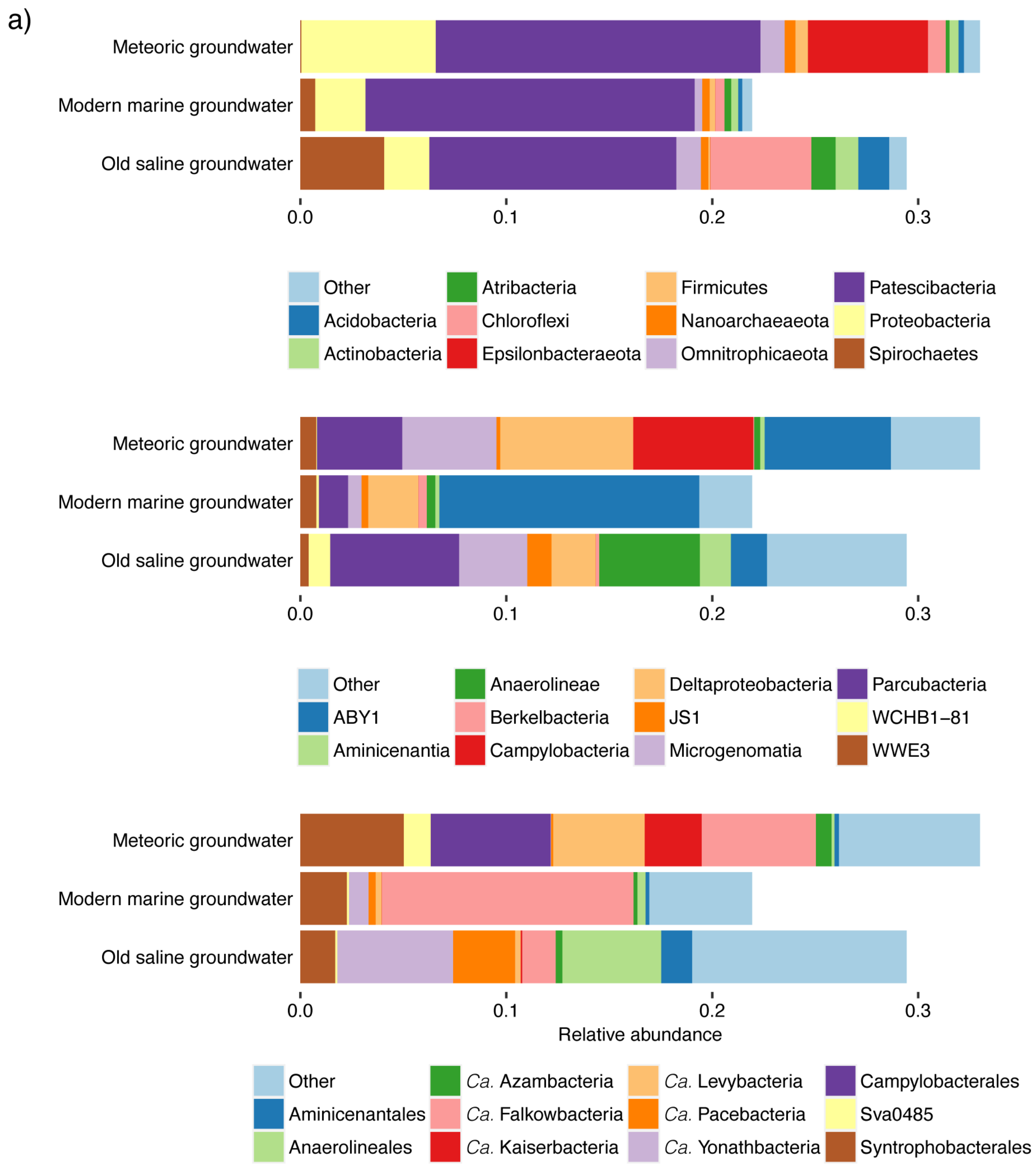

a)
Meteoric groundwater
Modern marine groundwater
Old saline groundwater
0.0
0.1
0.2
0.3
Other
Atribacteria
Firmicutes
Patescibacteria
Acidobacteria
Chloroflexi
Nanoarchaeaeota
Proteobacteria
Actinobacteria
Epsilonbacteraeota
Omnitrophicaeota
Spirochaetes
Other
Anaerolineae
Deltaproteobacteria
Parcubacteria
ABY1
Berkelbacteria
JS1
WCHB1−81
Aminicenantia
Campylobacteria
Microgenomatia
WWE3
Relative abundance
Other
Ca. Azambacteria
Ca. Levybacteria
Campylobacterales
Aminicenantales
Ca. Falkowbacteria
Ca. Pacebacteria
Sva0485
Anaerolineales
Ca. Kaiserbacteria
Ca. Yonathbacteria
Syntrophobacterales

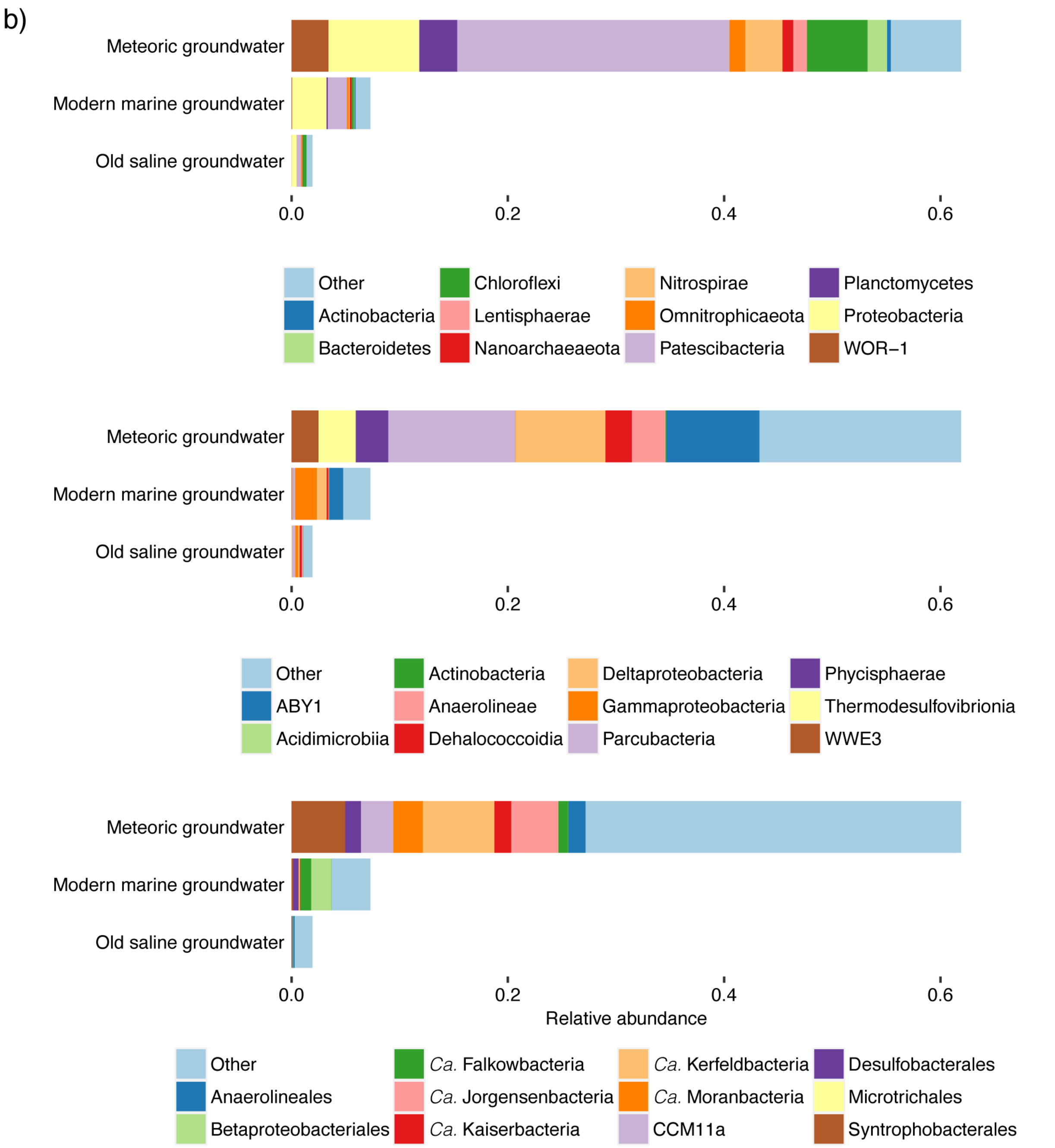

Fig. S5. Bar plots of the core and accessory community. The core (panel **A**) and accessory (panel **B**) community are sorted at the taxonomic level of phylum (top), class (middle), and order (bottom). The core microbiome constituted 24, 24, and 14% while the accessory microbiome constituted 67%, 6.0%, and 2.1% of the microbial community in the meteoric, modern marine, and old saline groundwaters, respectively. The figures depict the 11 most abundant groups over all the environments with the remaining taxa grouped as “Other”.
