# Supplementary material for: Connectivity of Fennoscandian Shield terrestrial deep biosphere microbiomes with surface communities
Source: Commun Biol. 2022 Jan 11;5:37. doi: 10.1038/s42003-021-02980-8 (PMC8752596; doi:10.1038/s42003-021-02980-8)
Supplement: Supplementary file 3 — Reporting Summary [file 42003_2021_2980_MOESM3_ESM.pdf]

nature portfolio

Corresponding author(s): G.P. Westmeijer

Last updated by author(s): November 24, 2021

Reporting Summary

Nature Portfolio wishes to improve the reproducibility of the work that we publish. This form provides structure for consistency and transparency in reporting. For further information on Nature Portfolio policies, see our Editorial Policies and the Editorial Policy Checklist.

Statistics

For all statistical analyses, confirm that the following items are present in the figure legend, table legend, main text, or Methods section.

n/a	Confirmed	
☐	☒	The exact sample size (*n*) for each experimental group/condition, given as a discrete number and unit of measurement
☐	☒	A statement on whether measurements were taken from distinct samples or whether the same sample was measured repeatedly
☐	☒	The statistical test(s) used AND whether they are one- or two-sided<br>*Only common tests should be described solely by name; describe more complex techniques in the Methods section.*
☒	☐	A description of all covariates tested
☐	☒	A description of any assumptions or corrections, such as tests of normality and adjustment for multiple comparisons
☐	☒	A full description of the statistical parameters including central tendency (e.g. means) or other basic estimates (e.g. regression coefficient) AND variation (e.g. standard deviation) or associated estimates of uncertainty (e.g. confidence intervals)
☐	☒	For null hypothesis testing, the test statistic (e.g. *F*, *t*, *r*) with confidence intervals, effect sizes, degrees of freedom and *P* value noted<br>*Give P values as exact values whenever suitable.*
☒	☐	For Bayesian analysis, information on the choice of priors and Markov chain Monte Carlo settings
☒	☐	For hierarchical and complex designs, identification of the appropriate level for tests and full reporting of outcomes
☐	☒	Estimates of effect sizes (e.g. Cohen's *d*, Pearson's *r*), indicating how they were calculated

Our web collection on statistics for biologists contains articles on many of the points above.

Software and code

Policy information about availability of computer code

Data collection	No software was used during data collection.
Data analysis	All software used in order to analyze the data was open-source. The figures in the manuscript have been produced in R Studio and the code has been uploaded to GitHub. The raw 16S rRNA sequencing reads were processed using Ampliseq with the reference provided in the manuscript.

For manuscripts utilizing custom algorithms or software that are central to the research but not yet described in published literature, software must be made available to editors and reviewers. We strongly encourage code deposition in a community repository (e.g. GitHub). See the Nature Portfolio guidelines for submitting code & software for further information.

Data

Policy information about availability of data

All manuscripts must include a data availability statement. This statement should provide the following information, where applicable:

- Accession codes, unique identifiers, or web links for publicly available datasets
- A description of any restrictions on data availability
- For clinical datasets or third party data, please ensure that the statement adheres to our policy

Data are available for the samples from nucleic acid sequencing repositories as detailed in Table S1. All data has been uploaded to public repositories. Part of the data was published prior to this study and (database) references are provided.

Field-specific reporting

Please select the one below that is the best fit for your research. If you are not sure, read the appropriate sections before making your selection.

☐ Life sciences ☐ Behavioural & social sciences ☒ Ecological, evolutionary & environmental sciences

For a reference copy of the document with all sections, see nature.com/documents/nr-reporting-summary-flat.pdf

Ecological, evolutionary & environmental sciences study design

All studies must disclose on these points even when the disclosure is negative.

Study description	The connectivity of deep biosphere groundwaters with overlying environments, focusing on the origin of the microbial communities residing in deep biosphere aquifers.
Research sample	Environmental samples from Baltic surface seawater, Baltic benthic seawater, two sediment depths, soil groundwaters and deep biosphere groundwaters (depth ranging from 70 m to 350 m). Geochemistry data on all groundwaters (soil, meteoric and deep biosphere).
Sampling strategy	Sampling in triplicates and using rarefaction curves to assess if full diversity is captured within a sample.
Data collection	For sampling details we refer to Table S2
Timing and spatial scale	Large variation of sampling dates. For example, Baltic surface seawater sampled between April 2011 and December 2013 (time series). This dataset was used as surface water takes several years (reference provided) to infiltrate underlying groundwaters and aquifers.
Data exclusions	Samples containing less than 1000 sequencing reads were removed from downstream analysis (3 out of 200+) as these failed sequencing.
Reproducibility	Sampling in triplicates, sampling various depths of sediment, soil and aquifers to capture maximum microbial diversity.
Randomization	Samples were categorized according to their environment of origin. Why these environments differed in their microbial composition was not the aim of this study.
Blinding	All samples were extracted using an identical protocol. Different environments were combined on one sequencing plate to reduce the effect of Illumina sequencing (sequencer, lane etc.)

Did the study involve field work? ☒ Yes ☐ No

Field work, collection and transport

Field conditions	Most of the field work involved sampling subsurface groundwaters and these were sampled under dry conditions with minimal temperature fluctuations.
Location	All samples were extracted from environments in South-East Sweden. Details are provided in Table S2 and a map of the locations in Fig. 2a.
Access & import/export	Collaboration with local organization maintaining the groundwaters and received assistance during sampling. Samples were immediately stored in liquid nitrogen in the field and stored in -80 upon arrival in the lab.
Disturbance	NA

Reporting for specific materials, systems and methods

We require information from authors about some types of materials, experimental systems and methods used in many studies. Here, indicate whether each material, system or method listed is relevant to your study. If you are not sure if a list item applies to your research, read the appropriate section before selecting a response.

Materials & experimental systems

n/a	Involved in the study
☒	☐ Antibodies
☒	☐ Eukaryotic cell lines
☒	☐ Palaeontology and archaeology
☒	☐ Animals and other organisms
☒	☐ Human research participants
☒	☐ Clinical data
☒	☐ Dual use research of concern

Methods

n/a	Involved in the study
☒	☐ ChIP-seq
☒	☐ Flow cytometry
☒	☐ MRI-based neuroimaging
